# A cardiologist’s guide to machine learning in cardiovascular disease prognosis prediction

**DOI:** 10.1007/s00395-023-00982-7

**Published:** 2023-03-20

**Authors:** Karl-Patrik Kresoja, Matthias Unterhuber, Rolf Wachter, Holger Thiele, Philipp Lurz

**Affiliations:** 1 Department of Internal Medicine/Cardiology, Heart Center Leipzig at University of Leipzig, Struempellstr. 39, 04289 Leipzig, Germany; 2 Leipzig Heart Institute, Leipzig Heart Science at Heart Center Leipzig, Leipzig, Germany; 3 Department of Cardiology, University Hospital Leipzig, Leipzig, Germany; 4 Clinic for Cardiology and Pneumology, University Medicine Göttingen, Göttingen, Germany; 5 German Cardiovascular Research Center (DZHK), Partner Site Göttingen, Göttingen, Germany

**Keywords:** Machine learning, Atherosclerosis, Heart failure, Arrhythmia, Genetics, Artificial intelligence

## Abstract

A modern-day physician is faced with a vast abundance of clinical and scientific data, by far surpassing the capabilities of the human mind. Until the last decade, advances in data availability have not been accompanied by analytical approaches. The advent of machine learning (ML) algorithms might improve the interpretation of complex data and should help to translate the near endless amount of data into clinical decision-making. ML has become part of our everyday practice and might even further change modern-day medicine. It is important to acknowledge the role of ML in prognosis prediction of cardiovascular disease. The present review aims on preparing the modern physician and researcher for the challenges that ML might bring, explaining basic concepts but also caveats that might arise when using these methods. Further, a brief overview of current established classical and emerging concepts of ML disease prediction in the fields of omics, imaging and basic science is presented.

## Introduction

A modern-day physician is confronted with a staggering increase of health care data created every day, surpassing the computational capabilities of the human brain by far [63]. While traditionally it has been the art of physicians to incorporate available data into clinical decision-making, this task seems to be unsolvable in the reality of the digital century. There is a broad consensus that artificial intelligence (AI) or machine learning (ML), often mistakenly used interchangeably [66], might pose a solution to this pressing issue. At the same time ML is seen as something unexplainable and unreachable by many health care workers. Soon, almost every physician will be using ML technology either consciously or weaved in the backend of medical software [76]. ML will likely aid us in diagnostic and management decisions, especially those that incorporate large digitized data [63]. In line with this, it is important for physicians and scientists alike to have a basic understanding of ML, to allow for a safe and conscious use in daily practice. The first part of this review will focus on providing a definition of ML and its capabilities, as well as limitations, while the second part will bring closer modern-day applications in cardiology including basic and translational science.

## Basic concepts of machine learning

The term ML was coined in 1959 by Arthur Samuel and refers to the idea that computer algorithms will learn and adjust and thereby improve automatically within their given boundaries through the use of data [67]. ML is considered to be a subset of AI and is therefore not an interchangeable term, and using it as synonym should be avoided. While ML and AI can clearly be distinguished from each other, it is quite hard to do so with conventional statistical methods [13]. Methods like hierarchical- or k-means clustering [31, 70] or linear regression [48] are increasingly termed as ML approaches. In fact, it is not easy to draw a clear line between conventional statistical approaches and ML [13]. In the absence of a uniform discrimination, both approaches can possibly be split according to their purpose: While conventional statistical models try to inform on relationships between variables in a mostly linear manner, ML is mainly focused on providing optimal predictions—often sacrificing interpretability [13]. There is no clear line to draw to distinguish conventional statistical models and ML. ML models for example can provide various degrees of interpretability ranging from the highly interpretable regularized regression method [56] to impenetrable neural networks, but in general, ML models will sacrifice interpretability for predictive power. In practice, this loss of interpretability is hard to accept, as the human mind is mainly used to understand linear associations between variables [30]. The interest in clinical ML and AI applications is increasing, yet it is important to keep in mind that there currently are no randomized clinical trials, which have shown that ML guided decision-making is superior to conventional approaches. However, ML represents a new chapter in science which has to be addressed, and by adding knowledge of ML methods to the toolbox of scientists and clinicians the black box can be opened and understood and future trials will determine the value of ML in clinical practice [27].

## Frequently used machine learning approaches

ML models can roughly be split into unsupervised and supervised approaches. While unsupervised ML focuses on discovering connections among variables, supervised approaches aim to achieve the optimal prediction of labeled samples (e.g. patients experiencing or not experiencing a defined outcome). Using unsupervised models, a classifier learns to infer relationships within the given data (e.g. to identify some clusters of patients who may carry the same genetic features of risk factors). Therefore, unsupervised ML can be of use to find new associations and suggest new hypotheses for new study designs, but its capacities in terms of predicting future events are limited. As this review focuses on prognosis prediction, we will focus the manuscript on supervised approaches and methodical concepts. Examples of unsupervised ML in cardiovascular medicine have extensively been reported before [25, 27, 32, 40, 44, 51, 63, 69–72].

**Fig. 1:**
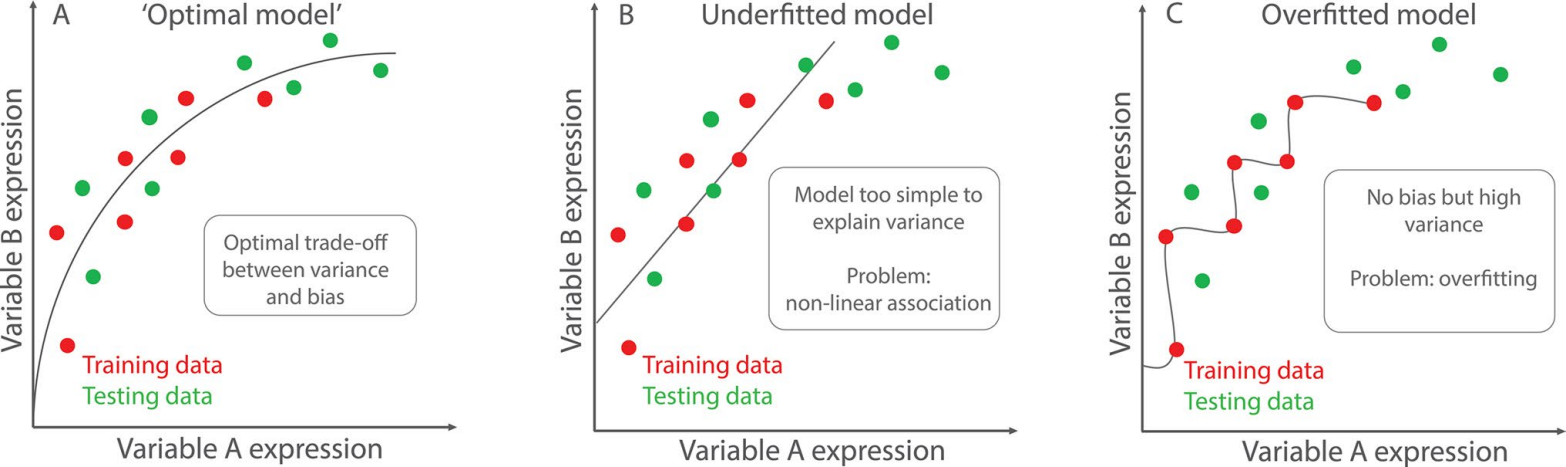
Examples of difficulties which might occur when choosing unfitting models. The figures represent examples of problems of under- (**B**) and overfitting (**C**) models. **A** Shows the example of an ‘optimal’ model, where an ideal trade-off between variance and bias is achieved. **B** Shows the example of an underfitted model. Albeit the data follow a non-linear relationship, a linear fit for the data was chosen. This often happens as linear regression might be preferred as the simplest solution to statistical problems, without acknowledging the true spread and association of data. **C** Shows the problem of overfitting, where the model is performing excellent on the training data, but struggles to predict unseen (testing) data. The model ‘learns’ the data rather than generalizable rules. Using machine learning solutions, one is usually rather prone to over- than underfitting, highlighting the importance of using external testing data to verify the generalizability of the model

Linear and logistic regression likely represent the most well-known statistical methods that can be referred to as ML approaches. They are hindered by a large number of assumptions, which are often violated in medical literature [22]. These include linearity, normality, homoscedasticity, as well as independence of data [82]. When these statistical assumptions are met, those models tend to perform well on training data but might struggle to make accurate predictions in never seen data, especially when linear assumptions are violated (Fig. 1). They tend to have low bias but high variance. For easier interpretation, a high bias is useful to accommodate human associative thinking and to discover correlations between independent and dependent variables, e.g., a biased model will tend to ignore outliers, creating a simple rule “valid for all”. This penalizes events with fine-grained influences which are unlikely to happen. A model with high variance starts from the assumption that relationships are complex and allows a model to assume many divergent predicting points. This will include more or less outliers for a certain degree, in turn it will not create straight lines, losing generalizability and interpretability. Further, this approach makes the model vulnerable to overfitting.

A possible way to improve the generalizability of a model is by performing so called regularized regression, which introduces a defined error termed λ. This penalization causes the model to perform a little bit worse on the training data but improves its prediction capabilities on unseen data [27]. The goal is to reach a favorable trade-off between the models’ decrease in accuracy and improvement in generalizability. Lasso regression can further be used to remove variables which add little to the overall model, which decreases the model’s complexity [15].

Supervised ML algorithms see a rapid increase in medical sciences as linear algorithms are often not capable to fully replicate biological reality [78]. For example, specific drug–drug interactions only might occur depending on the state of another variable [45]. Traditional statistical methods are limited in modeling such interactions, especially when considering a larger number of variables. One example are decision tree approaches which often can outperform traditional linear regression analysis on derivation, but due care must be given to not overfit models which will result in poor performance on validation data [18, 78]. Decision tree algorithms such as random forests [80], extreme gradient boosting or its predecessors adaptive boosting [83] are based on simple decision trees. Given a relevant number of variables, a normal decision tree can make a perfect prediction on a given dataset but will rather ‘memorize’ the shown dataset than learn generally applicable rules-the model ‘overfits’ (Fig. 1). The bias of such a model is zero, while the variance will be very high. Therefore, the aim in every ML decision tree algorithm is to reduce overfitting to avoid learning noise instead of generalizable patterns. Random forests were one of the first solutions described to address this problem. Instead of a very large and powerful ‘zero bias’ tree, random forests produce many smaller decision trees with data randomly sampled out and creation of random duplicates of the original dataset (‘bootstrapping’). Every produced tree could predict a different outcome for the same patient. Therefore, random forests do not provide a single answer but a ‘vote’, which comes down to the number of tree classifiers voting for either result this approach is often referred to as ‘bagging’ [11]. A further development of decision tree approaches are decision trees leveraging boosting methods. Again, ensembles are constructed from decision tree models, but unlike random forests, individual trees are not built on random subsets of data. Boosting approaches sequentially put more weight on instances with wrong predictions, so they learn from past mistakes. Trees are added one at a time to the ensemble and fitted to correct the prediction errors made by prior models. The latest development of boosting algorithms is the extreme gradient boosting (XGboost) approach recently developed by the University of Washington, which does not only outperform other decision tree approaches in terms of accuracy but has also an eighteen-times faster computing speed than random forests [16].

The last supervised ML approach we want to mention is deep learning (DL). DL aims to solve complex problems by mimicking the organization and functionality of the human brain with neural networks [27]. Nodes, which are labeled ‘neurons’ are arranged in a network layout. The first level of neurons feed into a finite number of other nodes called ‘hidden layers’ and can be considered as many layers of regressions. When a certain threshold of input is surpassed, a neuron in the hidden layer is ‘activated’ and by itself passes values further to neurons in the next layer. Like in linear and binomial regressions, these thresholds can be triggered by different values and follow straight, curved or edged lines. This goes on until the final layer is reached which is called output layer. DL excels at analyzing imaging data and are widely used for applications such as facial recognition or image enhancement. The training of DL networks require immense computational capacities, making their processing speed either very slow or very hardware demanding [27]. Reports of use of basic neural networks in cardiology data go as far back as to 1995 [6, 61]. Profound introductions into DL can be found elsewhere [7, 17, 24, 37, 65].

Table 1 shows a summary of advantages and drawbacks of the presented approaches to supervised ML.

**Table 1:** Examples of machine learning approaches

	Conventional regression	Regularization	Decision trees	Deep learning| neural networks
				
Examples	Linear regression Logistic regression	Ridge regression LASSO Elastic net	Simple decision trees Bagging Random forest Gradient boosting XGBoost	Convolutional neural networks recurrent neural networks long short-term memory networks
Model accuracy on linear data	↑	↑	↑	↑
Model accuracy on non-linear data	↓↓	↓↓	↑↑	↑↑
Model performance on imaging data	↓	↓	↓	↑↑↑
Interpretability	↑↑↑	↑↑	↓↓^a^	↓↓↓
User know-how required	↓	↔	↑↑	↑↑↑
Processing speed	↑	↑	↑	↓↓
Hardware requirements	↓↓	↓↓	↓	↑↑↑

^a^Interpretability is good for conventional decision tree models but becomes less when using bagging approaches with multiple decision trees incorporating votes on decisions

## Limitations of machine learning

ML is not the panacea for every unsolved and yet to come problem in medical sciences. As any other statistical model, ML models are limited by the quality and magnitude of signal in the dataset from which it is trained. The observation of an event is assumed to be the result of many causal factors. Theoretically, by knowing every predisposing factor and causal variable, an event could be perfectly predictable. In nature, however, adding variables to the equation can lead to noise due to measurement errors and methods, further many outcomes are associated to ever-changing non-predictable environmental factors (e.g., communicable diseases leading to infections triggering cardiovascular events). Even with the ‘perfect model’, the prediction can only be as good as the true connection between independent and dependent variables but in reality the unpredictability of external cues makes prediction of ‘all events and outcomes’ impossible [27]. So, while models are not able to provide an absolute truth, they are very good at providing probabilities.

Another practical problem is the quality of data used to train models. While omics analysis provides highly standardized and reproducible data, data from clinical practice might often be prone to higher variance alleviating relationships between outcome and input variables (e.g., NYHA class assessment). Imaging data also seem very standardizable at first thought, but there is a broad variety of different standards with respect to hardware and vendor software, making transferal of imaging data with sufficient quality often challenging. Lastly, there has been a broad discussion on a selection bias introduced into early established ML approaches used for facial image reconstruction due to a lack of diversity in the validation cohorts [49]. This emphasizes that more effort should be put on validation of established ML models in independent cohorts, rather than development of new approaches that only work well in the region or specific subset where it was developed.

Also of importance is the outcome the model seeks to predict. Mortality for example is an excellent outcome to predict as it is an absolute and discrete outcome, with an undoubtable definition. Other factors that are influenced by physiological variance like blood pressure measurements are more difficult to predict. Also, outcomes which require subjective definition might be challenging. While every endpoint adjudication committee will have no difficulties agreeing on whether a patient is dead, defining ‘simple’ categories such as cardiovascular death or heart failure hospitalization might become a point of disagreement. Accordingly, a study showed that a ML algorithm struggled to predict 30-day readmission for heart failure (C-index between 0.59 and 0.62) despite being fed with over 200 different variables accounting for demographics, socioeconomic status, medical history, characterization of heart failure (HF), admission and discharge medications, vital signs, weights, selected laboratories treatment, and discharge interventions [23]. Other studies have added to this demonstrating that ML models are better in predicting death than HF hospitalization given the same input data [53]. It is further important to keep in mind that there are outcomes that can be predicted well, but have only limited therapeutic use. In the clinical context, prediction should be focused on cases where actionable consequences for individuals arise.

Lastly, there is a disparity between the kind of answer provided by ML algorithms and the kind of answer required by physicians. Clinicians are expected to make a yes or no decision (e.g., initiate treatment or withhold), while ML algorithms provide probabilities (e.g., 64.73% chance of a patient responding favorably to initiating a specific treatment). A practical example can be found in everyday lab-charts. For example, high-sensitivity cardiac troponin T is usually presented with a cut-off of 14 pg/ml, which represents the upper limit of normal for a healthy reference population [33]. While for the physician it is very clear that a value of 15 pg/ml has a strikingly different risk than a value of 1400 pg/ml, guidelines have to provide decision-making support and break probabilities down into a ‘yes’ or ‘no’. This leads to a loss of valuable information and is called improper dichotomization [14, 28]. Dichotomization might become even less appropriate for lab values like hemoglobin, which might show U-shaped association with cardiovascular risk [57]. Therefore, physicians should keep in mind that most dichotomizations carry a significant loss of information and an informed physician will treat patients according to their personalized risk, as many already intuitively do [21, 27].

## The present use of machine learning in cardiovascular medicine

**Fig. 2:**
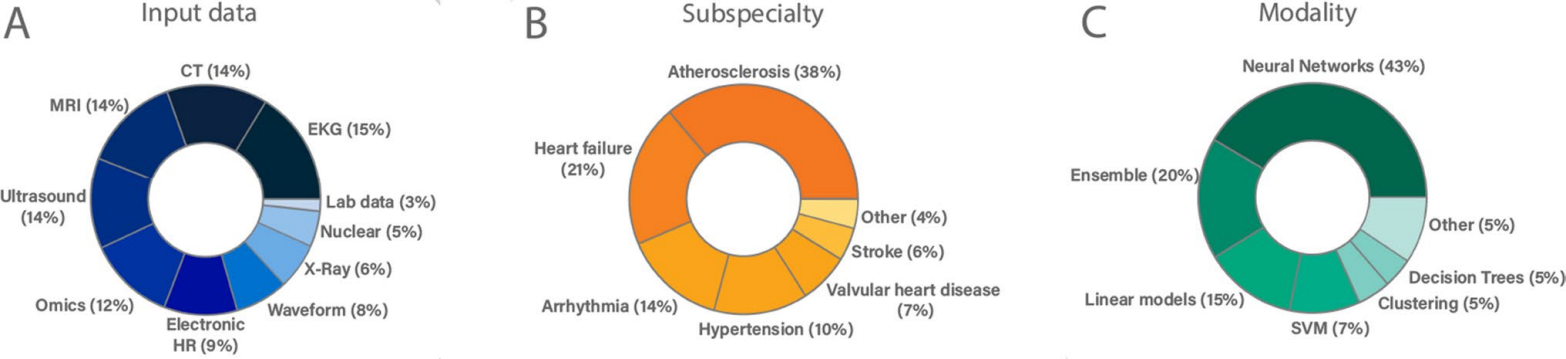
Current use of machine learning approaches in accordance to publications on Pubmed. Distribution of publications on machine learning according to **A** data types, **B** diseases and **C** ML methods used. Other includes basic science and congenital heart disease for the subspecialty section B and nearest neighbor method, as well as Gaussian and Bayesian analysis approaches for the modality section C. Data were mined by an algorithm kindly provided by Quer et al. [63]

**Fig. 3:**
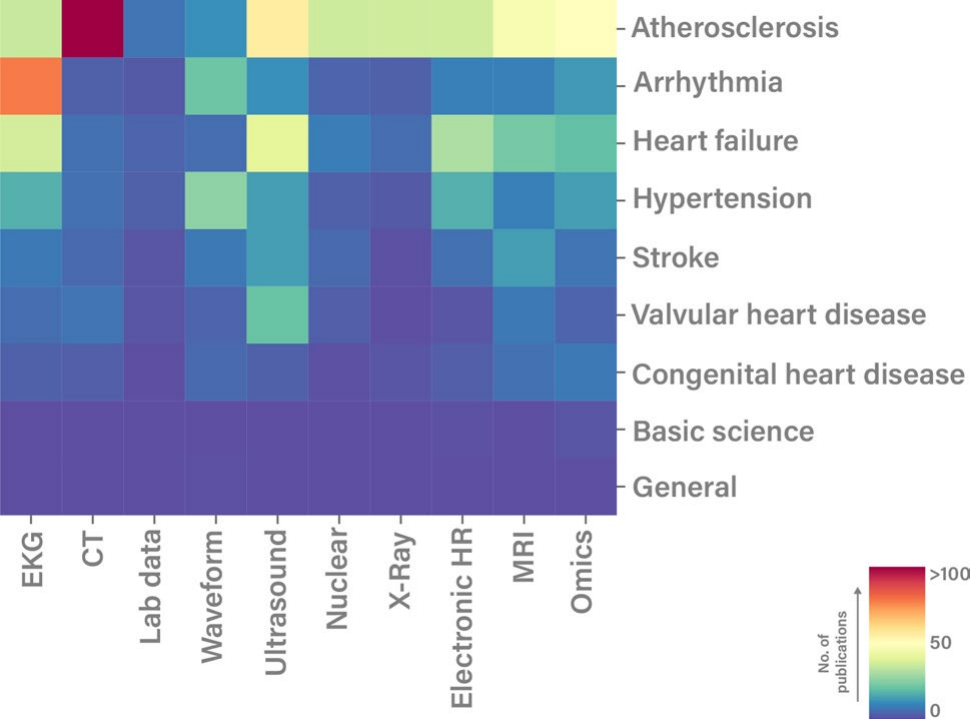
Heatmap of machine learning use in accordance to subspeciality by input data type. Heat map indicating the number of manuscripts with respect to disease and machine learning modality across the cardiovascular field. Data were mined by an algorithm kindly provided by Quer et al. [63]

Papers on ML have skyrocketed in the last years with over 80,000 Pubmed entries referring to either ‘machine learning’ or ‘artificial intelligence’. Using a miner algorithm kindly provided by Quer et al. [63] we displayed current uses of ML with respect to the source data used, the specific disease field investigated as well as the modes of ML used (Fig. 2). As shown, atherosclerosis is the main domain of ML use. Most of the input data used come from imaging like computed tomography (CT)- or magnetic resonance imaging (MRI) scans. DL makes up the largest fraction of ML models, which again is not surprising as they excel at interpreting these kind of imaging data [84]. Figure 3 shows a heatmap of the number of papers on ML in accordance to the subspeciality as well as input data used. Notably, basic science shows only a minor use of ML. In basic science understanding associations between variables might be more important than establishing strong but uninterpretable prediction models, yet there might be some promising new ML opportunities in this field discussed below.

## Prognostic value of machine learning in omics

The development of high-throughput platforms has left clinicians and scientists with a dilemma. It is now possible to analyze thousands of proteins, metabolites, genes, etc. with a minimum of material and effort. While statistical solutions have significantly developed over the last decade, they still have been outpaced by the rapid development seen in laboratory analytical approaches. This held true until the recent advent of ML approaches. ML enabled predictive algorithms now allow to better model the intricate working of physiology and pathophysiology alike [64].

Despite a vast number of established clinical risk scores for cardiovascular patients predicting outcomes among patients on an individual level remains challenging [38, 62]. The incorporation of biomarkers from large omics panels has shown promising results in the prediction of long-term mortality (C-index conventional cox regression 0.65 versus 0.93 for a XGBoost model, net reclassification improvement 78%) [78]. ML does not only improve long-term predictive capabilities but has also shown superiority in short-term prediction of mortality among patients with cardiogenic shock, by using biomarkers [15]. Performing advanced and importantly individualized risk prediction for patients might lead to intensified treatment in high-risk patients and therefore tailor optimal therapy for patients in dire need for optimized care [9]. But not only does ML enable the predictive capabilities of large omics data, it might also improve our understanding of these. By allowing for modeling of complex interactions, proteins are not forced into linear relationships and interpretative approaches like Shap values might be used to acknowledge the non-linearity of biological processes, or to identify possible novel treatment targets [79].

Amongst omics approaches, genetics represent one of the most growing fields in cardiovascular medicine and shows huge potential. Due to the goal of understanding pathways in disease-causing genetic disorders, ML has gained importance and is commonly used in genome-wide association studies (GWAS) [1]. ML methods have been successfully applied to predict the incidence of hypertension by using polygenic risk factors [39, 41, 58], to predict advanced coronary calcium [60], inheritable cardiac disease[12] and to predict type II diabetes in a multi-ethnic cohort [47]. Of interest, the number of layers within neural network architectures used in genomics has generally been far less than those used for image recognition [77], and typically consist of only a few layers [86] with many hundreds to thousands of parameters [35].

Another approach where ML might pave the way for cardiovascular medicine is the so called “liquid biopsy”. Liquid biopsy is a minimally invasive technology for detecting molecular biomarkers of a tumor without an invasive biopsy and has been established in oncology [8]. Liquid biopsy came up as a non-invasive way to characterize circulating biomarkers of tumor origin. Like in oncology we one day maybe will be able to fully characterize cardiovascular diseases such as heart failure, acute myocarditis, or coronary artery disease, without the need of further invasive testing and the combination of ML methods and multiomics approaches might be the way to achieve this ambitious goal [42].

## Prognostic value of machine learning in imaging

The capabilities in classifying objects in the entertainment and leisure sector started to improve with Kaggle competitions [10]. Up until today, image recognition abilities of neural networks are the most widespread application of DL in clinical practice. ML approaches might especially excel when analyzing standardizable two-dimensional images like electrocardiogram (ECG) data. For example, using a previously trained convolutional neural network in a prospective designed, non-randomized trial, ML was able to identify the occurrence of atrial fibrillation among patients at risk for a stroke with an odds ratio of 5.0 (95% confidence interval 2.3–5.4) [59].

For coronary artery disease (CAD), there is a large number of established ML algorithms for interpretation of CT scans and recently even approaches that aid decision-making and interpretation for invasive coronary angiography were proposed [54]. ML does not only aid in identifying patients with CAD but is also useful in prognosis prediction among these patients [2]. Using a mix of readily available clinical data and data from coronary artery CT scans Motwani et al. were able to show that ML outperforms conventional statistical models with regards to 5-year mortality prediction [55]. A combination of ML methods was presented within a prospective study of 1.912 patients with coronary artery CT scans, where extracardiac adipose tissue was quantified using a fully automated DL approach. Those data together with clinical variables, plasma lipid panel measurements, risk factors, coronary artery and aortic calcium scoring were analyzed by an extreme gradient boosting model and showed excellent predictive capability with respect to the occurrence of myocardial infarction and cardiac death [20]. Investigating approaches where different ML methods are combined on multiple levels could pave the way for an individualized patient risk assessment.

ML algorithms are also increasingly used to automatically interpret echocardiographic images and calculate factors like left ventricular ejection fraction to ease diagnosis of HF in clinical practice and have shown to outperform humans [5, 85]. (NCT05140642) These approaches might be leveraged to increase the interpretation as well as processing speed of lab animal echocardiographic assessment, increasing analysis speed while decreasing the need for additional workforce, after a stable workflow has been established.

## Prognostic value of machine learning in basic research

ML cannot only be used to predict clinical outcome but can also be leveraged to predict defined responses, or identify patterns previously missed by conventional approaches.

Most modern high-throughput genomics like Hi-C essentially represent a multilabel image classification problem and therefore DL approaches are ideal to address this problem. Recently, a DL framework called EagleC was presented to detect structural variations in human genome data. This algorithm was able to capture a set of fusion genes that are missed by whole-genome sequencing and was applied successfully to bulk and single-cell genomics in studying structural variation heterogeneity of primary tumors from Hi-C maps [81].

ML might enhance our understanding of biological processes when accurately dissecting them in logical units. Clerx et al. recently presented elegant ion-channel research, where they investigated the effects of mutations in the SCN5A gene on clinical phenotypes. New unclassified variants of SCN5A are regularly found but predicting their pathogenicity has proven exceedingly difficult. Clerx et al. established a two-step ML model which first aims to establish effects from the gene to functional properties (from gene to function) and then adds a second step where predicted functional properties are used to describe a clinical phenotype (from function to phenotype). They were able to show an improvement in the prediction of functional sodium channel properties and outperformed traditional approaches with the prediction of clinical phenotypes, but predictive performance remained limited [19]. The strength was to split the process into a multi-step approach, which is often the case in biological reality, but the approach was limited due to the imbalance of the available data favoring pathogenic mutations. This practical example highlights the importance of quality of both the outcome as well as the input data used for ML models.

DL has also been described to excel in aiding the analysis of cellular imaging. Beneficial use with image classification, segmentation, object tracking and augmenting microscopic images has been demonstrated. DL for these applications is still in its early phase but has shown promising results and are positioned to render difficult analyses routine and to enable researchers to carry out new, previously impossible experiments [52].

Recently, AlphaFold 2, a ML software developed by Alphabets DeepMind company, has been acknowledged as a milestone in the problem of protein folding [29]. As a protein’s physiological function is determined by its three-dimensional structure, the knowledge of latter is essential to understand the biological processes involved [68]. Protein folding is hard to forecast and while models based on simulating quantum mechanics showed promising results, calculation of protein folding in large proteins is challenging due to the exponential rise in required computational capacity. Fundamentally, protein folding is an imaging challenge. It was, therefore, speculated that ML, especially DL, might perform very well. In fact, AlphaFold 2 using novel deep learning approaches, has altered the field of protein folding prediction, vastly outperforming all other 145 presented approaches at the CASP14 competition (Critical Assessment of Structure Prediction, a bi-annual competition for the prediction of protein folding) [73]. AlphaFold 2 as an open-source end-to-end user software is expected to have a relevant impact on the field of protein folding prediction making it more time efficient and accessible for public. However, it is important to keep in mind that, while encouraging, AlphaFold 2 has the limitation that it was trained on the protein data bank (PDB), which incorporates many protein structures which were observed only during experimental conditions, which might not reflect biological reality. Therefore, while AlphaFold 2 is a masterpiece of ML programing, as any other model, it is not protected from the bias of the data used to train the model. Ultimately, ML aims at prediction or reflecting reality, with the latter is always difficult to define and derive [46].

Lastly, ML can also be used to inform on pathways involved in outcomes of interest following steps undertaken by specific peptides, all the way up to their origin in the genome [79]. Jaganathan et al. successfully trained a DL algorithm to predict splicing from pre-mRNA out from a genomic sequence to detect noncoding mutations in rare genetic diseases, possibly paving the way for a reverse engineering of the human genome-protein processing [26]. Further, ML was used for designing molecules to modify specific targets, but also to influence properties like solubility and bioactivity, raising the level of this former trial and error approach to a precision process [75]. In the near future, ML approaches might, therefore, allow treatment response prediction, assessment of in silico protein interactions, identification of novel drug targets, monitoring and predicting response to latter; all of these leading towards a patient-tailored precision medicine approach. There are already interesting commercially available systems like IBM Watson Health’s cancer AI algorithm, an algorithm used for recommending treatments for patients with cancer trained on simulated cases. However, the algorithm has been shown to give erroneous outputs in some real-life cases underlying the importance of proper input data and prospective validation [76].

## How to interpret ML applications as clinician

**Fig. 4:**
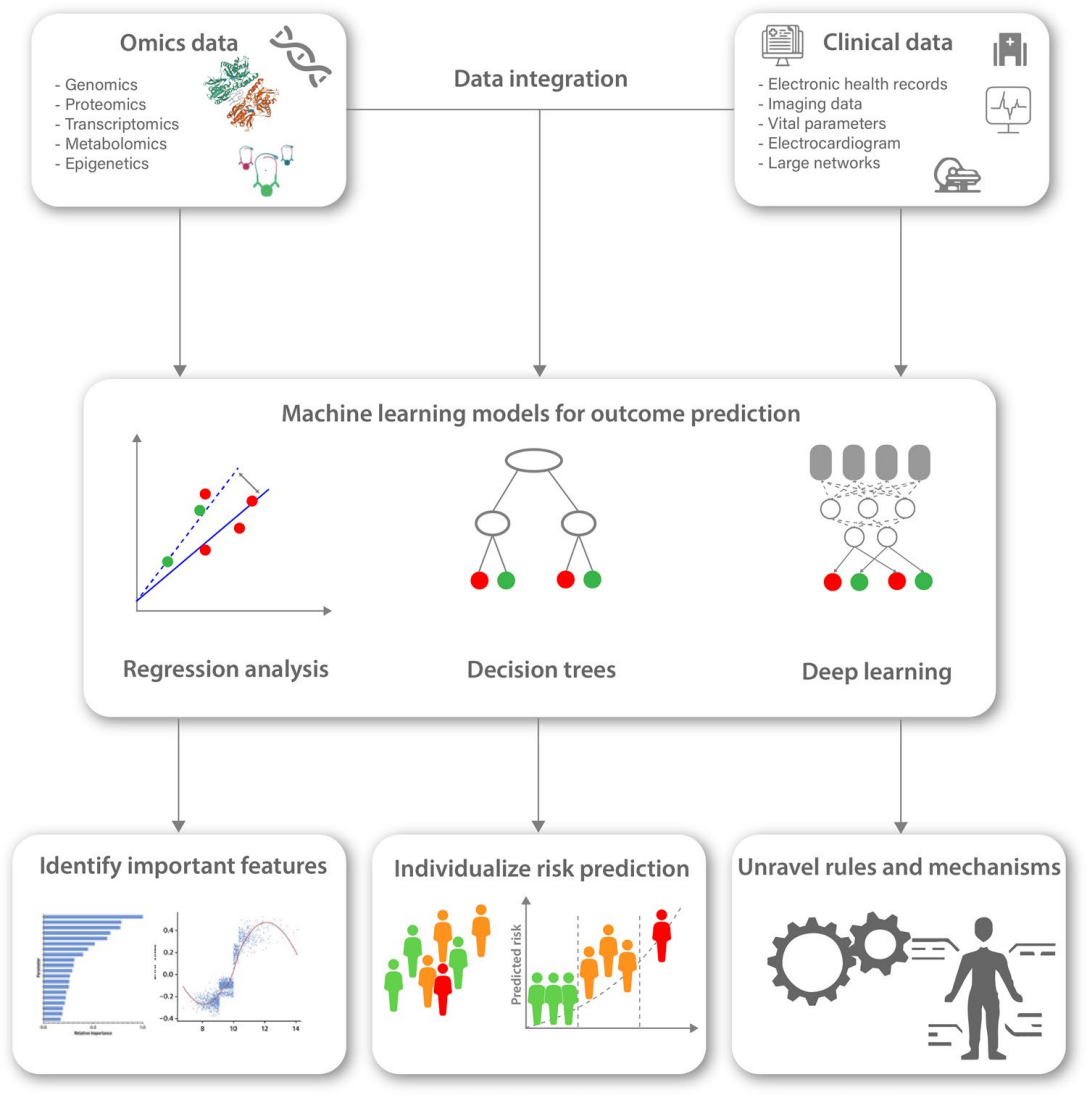
The use of machine learning models to integrate complex multi omics data as well as clinical data and their potential to support clinicians and scientists. Machine learning approaches have the potential to integrate large numbers of variables from large populations to allow for individualized risk prediction. This can be translated into clinical practice by reporting on important features, establishing clinical phenotypes with comparable outcomes and ultimately identifying novel pathomechanisms

Cardiovascular medicine is well positioned to leverage ML methods to facilitate precision medicine approaches by integrating the vast abundance of ‘omics’ and clinical data available (Fig. 4) [4, 76]. Scientists and clinicians alike often reject ML approaches as they argue that they are a ‘black box’ and the predictions made by ML are not understandable [50]. Yet, in practice we rely on many established scores and predictors which we do not truly understand. In fact, most scientists and clinicians will acknowledge that they do not know the derivation and validation of a large fraction of scores they use. While they might be easy to calculate without the knowledge of how they were derived and validated the trust we lay upon them is in fact no lesser a ‘black box’ than the trust we may lay upon ML. It is important to keep in mind that interpretability might mean different things for clinicians, scientists but also patients [36]. Interpretability by itself might also have different levels. Van der Schaar proposed 3 types to provide interpretability for ML models. The first one is to show explanatory patient features. This means highlighting what features were considered in the ML model and how they were weighted. Gain plots and SHapley Additive exPlanations (SHAP) values provide practical example of this application [43]. The second type suggests grouping patients according to similarities with regards to the predicted outcome, this might allow to identify specific high- or low-risk phenotypes. The third type is vastly harder to establish as it suggests identifying rules and laws, that cause the model to significantly alter a decision and therefore reduce the model to the most important decisions. The first and second types are already practical reality and used in many papers, but while they seem to thoroughly inform on the model they might also introduce dangers as they do not allow on the causal relationship of outcome and the variables of interest. The third type which allows the establishment of rules might likely be the most sought-after approach in clinical practice, but up until today no valid method exists to establish these laws.

To be able to interpret new ML solutions, it is essential for physicians to apply structured criteria. In line with this the U.S. Food and Drug Administration (FDA), as well as the Standards for Reporting of Diagnostic Accuracy Study (STARD) have established standard of ‘Good machine learning practice’ and STARD-AI guidelines, respectively, to allow for a common scientific standard [74]. In line with this we provide an overview of questions inspired by Meskó et al. [50] that physicians can ask themselves when assessing new ML approaches in Table 2.

**Table 2:** General questions to ask when assessing new machine learning models in clinical practice

Issue	Important questions to ask
Relevance of the machine learning model	Does the ML model provide incremental value over established non-ML models? Does the predicted outcome have actionable clinical consequences or other relevance? (either for patients and/or relatives)
Sample size and validation	Does the ML model study provide a validation cohort? Are the sizes of the derivation/training and validation datasets justified? Are there sufficient overall cases to justify a ML approach at all? (e.g., Orphan diseases)
Selection bias	Is the investigated cohort representative for my patient population? Do the incidences/events observed reflect real-world numbers? Will the model be applied to comparable situations as it was developed in?
Labeling outcomes	How were outcome labels defined? Does the labeling used reflected the current gold-standard in the specific field? Are the labels standardizable and transferable to other cohorts? (e.g., omics data) Are there differences between imaging software/hardware/vendors that hinder easy integration of data sets?
Guideline adherence	Does the ML algorithm development and reporting follow the U.S. Food and Drug Administration (FDA), or the Standards for Reporting of Diagnostic Accuracy Study (STARD) guidelines? Was there a prespecified analysis plan published?
Transparency	Is there supporting information on how the ML model derives its decisions? Is the ML model publicly available? Are there monetary/patent interests of the authors or other groups on the presented ML algorithm?

## Outlook

ML poses a vast amount of opportunities, but this does not come without the cost of certain pitfalls which have been discussed within the first part of this review. In cardiology, ML has already reached center stage and many fields have already seen interesting proof-of-concept studies especially as medical data is increasing in amount and complexity daily. However, it is important to keep in mind that ML approaches are not always the optimal solution and especially in basic research where causal associations between variables might be more important than optimized predictions, conventional statistical approaches might at this stage provide better use than modern ML techniques. But acknowledging the potential of ML methods which allows non-linear assessment of associations, that further accounts for complex interactions, ML might be able to help basic research methods raise to new frontiers in a large scale of fields, especially in the setting of high-throughput data like omics. Further, ML approaches might be used to streamline image processing [34] and, therefore, increase and standardize workflows traditionally prone to require high amounts of work force.

In the light of the complex interplay of variables used in ML, surpassing the capabilities of the human brain, it will be more important than ever to validate those algorithms meticulously in prospective clinical trials [3]. After all, even if we may not be able to grasp the individual calculations the ML algorithm makes, we must be able to trust in the answers it provides.
